# The Comparative Effect of Acute Moderate- and High-Dose Citrulline Malate on Resistance Exercise Performance in Trained Individuals: A Double-Blind Randomised Controlled Pilot Trial

**DOI:** 10.3390/jfmk11010115

**Published:** 2026-03-10

**Authors:** Lewis A. Gough, Rachel Tan, Stephen J. Bailey, Craig Perrin, Charlie J. Roberts, Freya Gibbons

**Affiliations:** 1 Research for Human Performance and Health Laboratory, Centre for Life and Sport Science, Birmingham City University, Birmingham B5 5JU, UK; 2 Department of Sports Medicine, Pepperdine University, Malibu, CA 90263, USA; 3 School of Sport, Exercise and Health Sciences, Loughborough University, Loughborough LE11 3TU, UK

**Keywords:** supplements, nitrate, dose–response, exercise

## Abstract

**Background:** Citrulline malate (CM) supplementation has been shown to improve resistance exercise performance. However, there is limited research on the dose–response effects of CM ingestion. The aim of this study was to investigate a moderate (8 g; CM-MOD) and high (12 g; CM-HIGH) dose of CM on resistance exercise performance. **Methods:** Twelve resistance-trained individuals (7 females, 5 males, age = 24 ± 2 years; body mass = 70 ± 10 kg; height = 172 ± 7 cm) volunteered for this randomised, double-blind, crossover trial. Following a familiarisation trial that consisted of determining one repetition maximum, participants completed barbell bent-over rows and leg presses following acute ingestion of either 8 g CM (CM-MOD), 12 g CM (CM-HIGH), or a placebo 1 h prior to exercise. Each exercise comprised two sets of 10 repetitions (70% one-repetition maximum (RM)) and a third set to exhaustion at 70% 1 RM. **Results:** The linear mixed-effect model found no significant differences in the completed repetitions between exercise type but did reveal a significant main effect of CM-HIGH on repetitions completed (*p* = 0.032), which was not found for CM-MOD, and only increases in leg press repetitions were observed (estimated marginal means: placebo = 17; CM-MOD = 19; CM-HIGH = 20). **Conclusions:** In conclusion, CM-HIGH resulted in small improvements to total repetitions performed during resistance exercise performance and likely only during leg press activity, though the underlying mechanisms remain unclear and further investigation is warranted.

## 1. Introduction

Citrulline malate (CM) is an inorganic salt synthesised from the non-essential amino acid L-citrulline and the tricarboxylic cycle intermediate malate. As a precursor for L-arginine, L-citrulline ingestion can augment the synthesis of nitric oxide, a key signalling molecule that facilitates vasodilation [1]. Combined L-citrulline and malate consumption has also been shown to increase blood flow to skeletal muscle, which has been largely evidenced by a reduction in post-exercise systolic and diastolic blood pressure [2]. Malate may also have additional mechanisms including increasing the rate of oxidative ATP production and phosphocreatine re-synthesis [3]. Furthermore, the addition of malate may increase the elimination of ammonia produced during high-intensity exercise, as inferred by high post-exercise lactate, and increase tricarboxylic cycle efficiency via enhancement of the malate aspartate shuttle (MAS) [4]. Based on these mechanisms, CM ingestion is potentially well suited to resistance exercise performance on the premise that these mechanisms would lead to increased training output, and this could increase the potential strength and/or power improvements from training.

Positive findings have been reported following acute CM ingestion with the commonly employed dose of 8 g when consumed 30–60 min prior to exercise [5,6,7]. These collection of studies have reported improvements in the number of repetitions performed to failure, a reduced rate of perceived exertion and/or delayed-onset muscle soreness. For example, Pérez -Guisado and Jakeman (2010) [5] reported total reps during four sets to failure (at 80% 1RM) were improved significantly following ingestion of 8 g CM. Unfortunately, out of the studies reporting benefits only Wax et al. (2015) [6] reported post-exercise lactate and this was unchanged, which makes it difficult to draw conclusions on the acting mechanism. They also reported no significant difference in post-exercise blood pressure, which would allude to a non-blood flow mechanism. Moreover, these effects are not universal across studies, with many reporting no effects of CM ingestion on resistance exercise protocols [8,9,10]. Reasons for these discrepancies could have been the known issues with supplement quality, where the citrulline:malate ratio in the CM administered to participants via independent testing was much lower than purported by the manufacturer (manufacturers claims: 2:1 vs. independent testing: 1.1:1) [8,9]. Equally, there is variation in the methodologies across studies, including the percentage RM used in the studies, exercise selection, and participant training status. Given the varied approaches in the literature to date, further research is required.

There are implications for training if improvements in total repetitions performed are observed following CM ingestion. Specifically, training volume (i.e., repetitions × load) is correlated to hypertrophic gains, in that the higher the volume the greater the potential hypertrophy [11]. The greater training volume may also increase mTOR signalling, VEGF expression and therefore angiogenesis, and endothelial signalling [12]. There are limited studies currently on the precise mechanisms; however, post-exercise lactate could be used as a proxy marker for metabolic stress. As CM is associated with lower post-exercise lactate, possibly through ammonia clearance, it is not currently clear if this may counteract the potential adaptation benefits from the increase in training volume or enhance it via improving overall training volume.

Dose–response data from Moinard et al. (2008) [13] suggest that doses of citrulline alone at 15 g maximise the citrulline response without adverse side effects and acceptable tolerance [14], compared to lower doses such as 2 g, 5 g and 10 g of citrulline [13]. Given that increase citrulline will lead to increase NO bioavailability, and the latter has been associated with increased performance responses, it is postulated that this would enhance performance further [15]. In addition, this may explain the mixed performance outcomes following CM ingestion reported in the literature as commonly used dosesmay be suboptimal. However, there have been no studies examining the potential impact of a higher CM dose on resistance exercise performance or physiological responses (i.e., lactate, heart rate, blood pressure). Therefore, the aim of this study was to investigate the effects of moderate (8 g) and high (12 g) doses of CM supplementation compared to placebo on resistance exercise performance. The hypothesis of the study was that only the high dose of CM would significantly improve resistance exercise performance.

## 2. Materials and Methods

### 2.1. Participants

Twelve resistance-trained participants were recruited for this block-randomised (3-block design), double-blind, crossover pilot study (7 females, 5 males, age = 24 ± 2 years; body mass = 70 ± 10 kg; height = 172 ± 7 cm; Table 1). Participants met the classification for tier 2 (trained/developmental) as described by McKay et al. (2022) [16]. A sample size calculation was not conducted due to the pilot nature of the study and the fact that there was limited exercise data to determine our required effect size. Finally, the sample size was primarily determined by resource constraints. Four visits were required, consisting of one familiarisation and three experimental trials, where either 8 g, 12 g or a placebo was ingested (order block-randomised). The inclusion criteria required participants to be ≥21 years old, free of injury in the six months prior to the start of experimental testing and engaging in regular self-reported resistance training that featured leg press and barbell bent-over row exercises (≥3 h per week). Participants had to have completed this type of exercise for a minimum of 1 year. Female participants were tested within the luteal phase of the menstrual cycle. All participants provided voluntary informed written consent, and the study received institutional ethical approval (Gibbons/#12761/sub2/R(B)/2024/Apr/HELS FAEC). The study was conducted in accordance with the appropriate ethical standards as presented in the 1964 Declaration of Helsinki and its later amendments.

### 2.2. Preliminary Procedures

Prior to attending a familiarisation session, participants completed a Physical Activity Readiness Questionnaire and underwent anthropometric measurement testing for height (SECA stadiometer SEC-225, SECA, Hamburg, Germany) and body mass (SECA digital column scale SEC-170, SECA, Hamburg, Germany). Additionally, participants were asked to provide a 24 h written food diary before the familiarisation session and were then required to replicate this prior to each subsequent trial. This was confirmed verbally prior to each trial. They were also asked to avoid mouthwash and supplements containing NO. Each written log was inspected prior to each trial to ensure adherence to the study procedures. Experimental trials were conducted at the same time of day (±1 h) to account for circadian rhythms, with ≥7 days separating each trial. Furthermore, participants were asked to refrain from consuming caffeine-containing products within 12 h of experimental trials and to avoid exhaustive exercise 24 h prior to each trial.

During the familiarisation session participants were informed of the experimental procedures. One-repetition maximum (1RM) testing was then conducted for barbell bent-over row and leg press exercises, with the latter exercise completed on a commercially available device (Selection 900 Leg Press, TechnoGym, Bracknell, UK). Once determined, participants completed a small number of reps (5–10) to familiarise themselves with the calculated weight. These exercises were selected as the participants were already completing these exercises regularly. The barbell bent-over row featured a 20 kg barbell (Eleiko, Halmstad, Sweden) and plates (TechnoGym, Bracknell, UK) with use of both arms. Each exercise was performed as per the guidance in Baechle and Earle (2014) [17]. For each exercise, participants were asked to warm-up using resistance allowing for the completion of 5–7 repetitions. Following a one-minute rest period, additional resistance was added (bent over row: 4–9 kg; leg press: 14–19 kg) and participants were instructed to complete 3–5 repetitions. This protocol was repeated until participants were unable to complete a single repetition with correct form. The heaviest load lifted successfully was then determined as the 1RM.

### 2.3. Experimental Procedures

**Figure 1:**
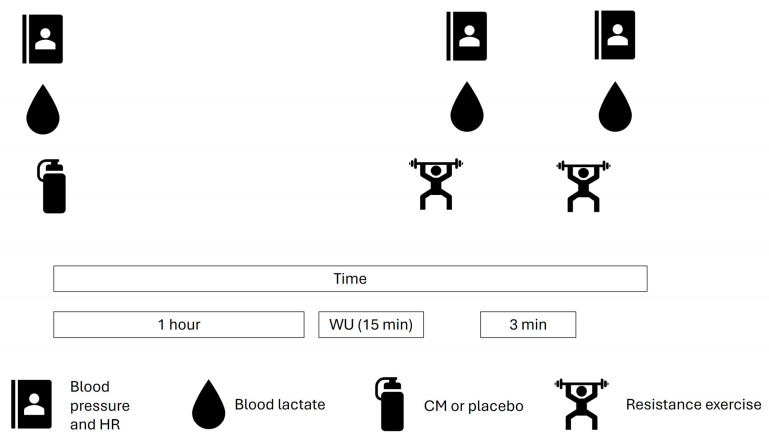
Schematic of experimental trials. HR = heart rate, CM = citrulline malate, min = minute.

An overview of experimental trials is depicted in Figure 1. At baseline, systolic and diastolic blood pressure (BoSo-Medicus, Bosch and Sohn, Jungingen, Germany), blood lactate (Lactate pro 2, Arkray, Kyoto, Japan), and heart rate (Ignite 2, Polar, Kempele, Finland) were measured after a 5 min seated rest. Blood lactate was sampled via a 5 μL finger prick capillary blood sample.

Participants were then provided with non-transparent water bottles containing either 8 g (CM-MOD) or 12 g (CM-HIGH) CM solutions, or a taste-matched placebo. CM solutions contained the supplement (Bulk, Henderson, NV, USA) mixed with 100 mL soda water (Morrisons Supermarkets Ltd., Bradford, UK) and 25 mL lime cordial (Morrisons Supermarkets Ltd., Bradford, UK), with the placebo solution consisting of 100 mL of soda water and 50 mL lime cordial. Soda water was selected to mimic the fizz produced by CM, with pilot testing confirming this to be an adequate blinding procedure (taste, smell, and appearance). Solutions were mixed by a laboratory technician not involved in the study and were not seen or handled by any of the research team (authors). Participants were instructed to ingest the solutions within three minutes ~1 h prior to exercise, which was based on the time to peak concentration of citrulline [13]. Furthermore, an 8 g dose was selected as this is the most common dose in the literature and it would also offer a comparison to the higher dose. Following ingestion of the solution and a 45 min rest period, participants were instructed to engage in a self-selected 15 min warm-up, with individual selections recorded and replicated for each experimental trial. Generally, the warm-up consisted of completing a self-selected number of repetitions and sets for barbell bent-over rows and leg presses at ~30% 1RM (generally 10–15 reps and 1–2 sets).

Across each experimental resistance exercise set, participants started with barbell bent-over rows. Participants performed two sets of 10 repetitions at 70% 1RM interspersed with three-minute rest periods between sets. This was followed by a third set to failure where total repetitions were the main outcome measure of the study. Failure was determined by the lead researcher calling failure at the instant a repetition that was not performed using a full range of motion or acceptable form. Participants were then seated for three-minutes for re-assessment of systolic and diastolic blood pressure, blood lactate, and heart rate. The same measurements (blood pressure, lactate, and heart rate) were then replicated for the leg press exercise and the order of exercises was consistent throughout the study (i.e., barbell bent-over row followed by leg press).

### 2.4. Statistical Analysis

Statistical analyses were conducted using R statistical software (version 4.3.3; R Core Team), primarily using the lme4 package. A linear mixed-effect model was used to compare effects of citrulline malate dosage (0 g, 8 g and 12 g) at three time points (pre-, mid-, and post-RT) for each physiological response (lactate, heart rate, systolic and diastolic blood pressure). In addition, a linear mixed-effect model was used to compare the effects of citrulline malate dosage (0 g, 8 g and 12 g) and exercise type (leg press or rows) on the number of repetitions completed. For all models, CM dosage and either time or exercise type were treated as fixed effects, while a “participant” factor was included as a random intercept to account for the inter-individual variability. For each model, the assumptions of normality and homoscedasticity of residuals were checked visually. Homoscedasticity was evaluated by plotting residuals against fitted values, and normality was assessed using Q-Q plots of the residuals. For lactate, residuals displayed moderate right skewness with larger residuals at higher values of lactate. Therefore, lactate data was log-transformed. Multicollinearity among fixed effects was assessed using Generalised Variance Inflation Factors (GVIF). All adjusted GVIF values were below two, indicating no concerning multicollinearity. Estimated marginal means (EMMs) and pairwise comparisons were computed using the emmeans package with Tukey adjustments to interpret significant main effects and interactions. Statistical significance was set at *p* < 0.05 for all comparisons.

## 3. Results

**Figure 2:**
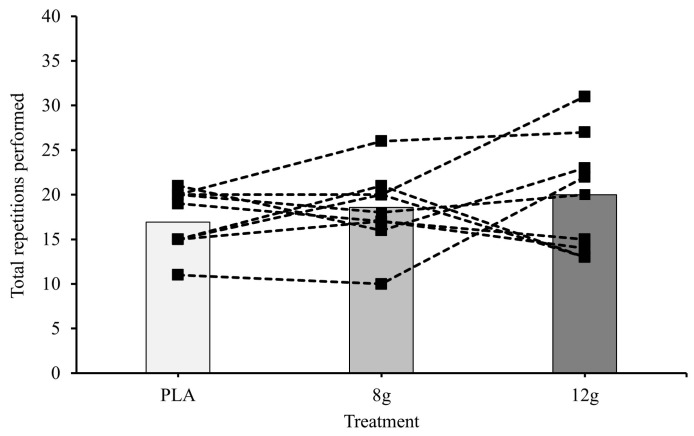
Mean and individual responses to placebo (PLA), 8 g (CM-MOD), and 12 g (CM-HIGH) for total repetitions during leg press. Error bars omitted for clarity.

**Figure 3:**
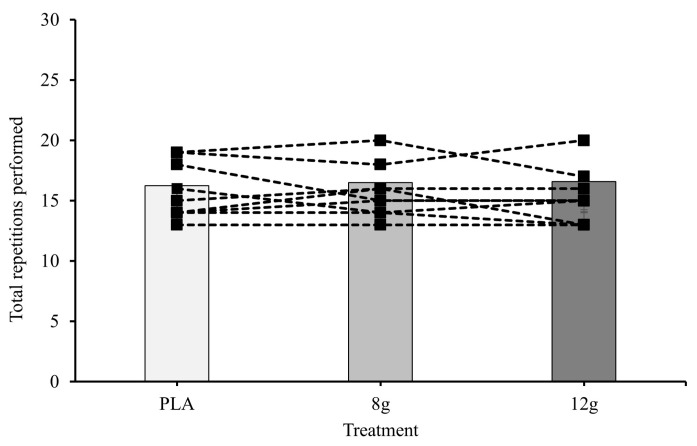
Mean and individual responses to placebo (PLA), 8 g (CM-MOD), and 12 g (CM-HIGH) for total repetitions during bent-over row. Error bars omitted for clarity.

The linear mixed-effect model found no significant differences in the completed repetitions between exercise type but did reveal a significant main effect of CM-HIGH on repetitions completed (*p* = 0.032), which was not found for CM-MOD. However, post hoc testing was unable to find a significant difference in repetitions between CM-HIGH and CM-MOD or placebo (20, 18, and 17 repetitions respectively). However, exploratory analyses indicate a possible interaction between CM dosage and exercise type. Although not significant, estimated marginal means show an increase in leg press repetitions from 17 at placebo to 19 at CM-MOD and 20 at CM-HIGH, whereas repetitions in the row exercise remain consistent across dosages (EMMs were within 0.4 repetitions; Figure 2 and Figure 3).

**Figure 4:**
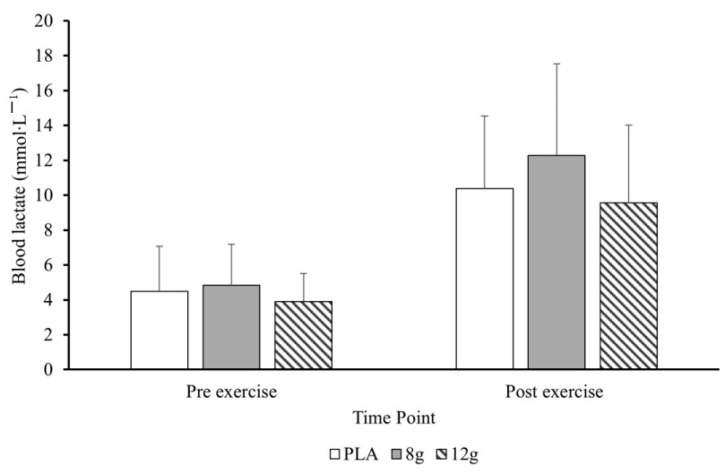
Mean ± standard deviation blood lactate from pre- to post-exercise leg press following placebo (PLA), 8 g (CM-MOD), and 12 g (CM-HIGH).

**Figure 5:**
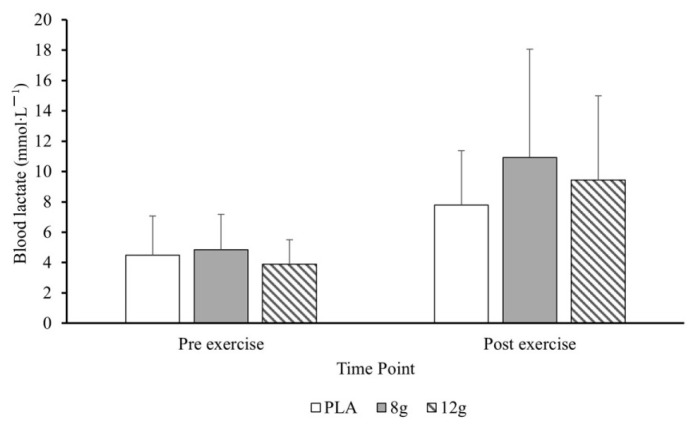
Mean ± standard deviation blood lactate from pre- to post-exercise bent-over row following placebo (PLA), 8 g (CM-MOD), or 12 g (CM-HIGH).

Mean pre- and post-exercise blood lactate responses for leg press and bent-over row are displayed in Figure 4 and Figure 5, respectively. Lactate significantly increased at the Middle and Post RT (*p* = 0.003 and *p* < 0.001, respectively). Post hoc pairwise comparisons using emmeans confirmed lactate was significantly higher at Middle (7.92 mmol/L; *p* < 0.001) and Post (9.71 mmol/L; *p* < 0.001) compared to Pre (3.89 mmol/L), with no significant difference between Middle and Post (*p* = 0.21). Similarly, heart rate significantly increased Post RT (*p* = 0.027) but not at Middle (*p* = 0.193). Post hoc pairwise comparisons using confirmed HR was significantly higher at Post RT (85 beats·min^−1^; *p* < 0.003) compared to the Pre (75 beats·min^−1^), but not at Middle (82 beats·min^−1^; *p* = 0.576). There were no significant effects of citrulline malate for lactate or heart rate.

Time had no significant effect on diastolic blood pressure. CM-MOD reduced diastolic blood pressure (*p* = 0.049), though significance was not achieved with CM-HIGH (*p* = 0.099). For systolic blood pressure, there was a significant interaction between CM-HIGH and the Middle of RT. However, post hoc contrasts did not reveal significant difference between CM dosages at any time point compared to Pre (Middle: *p* = 0.169; Post: *p* = 0.6065).

## 4. Discussion

The aim of this study was to assess the effects of CM-MOD and CM-HIGH on resistance exercise performance. The results of this study show that CM-HIGH may improve total leg press repetitions, whereas CM-MOD and placebo revealed no significant effects. For barbell bent-over rows, no significant effects were reported following ingestion of either CM dose. The findings of this study may therefore support the use of CM-HIGH for improving total repetitions during resistance exercise performance, specifically during leg press activity. Future research should explore CM-HIGH with larger sample sizes and investigate the effects of chronic ingestion on training outcomes.

CM-HIGH improved the total number of repetitions of leg press and CM-MOD was not ergogenic for either exercise, and these findings with the latter dose of CM agree with other studies that found no effects of CM ingestion on resistance exercise performance [7,8,9]. This is likely due to the dose of 8 g CM, as the current study reported ergogenic effects with CM-HIGH only. The latter dose would have increased plasma citrulline in line with peak concentrations [13] and this may explain why an ergogenic effect was reported. It is worth noting, however, that the ergogenic benefits with CM may be due to the exercise intensity selected in the current study, as studies with 8 g CM that report ergogenic benefits were at a much higher exercise intensity. Specifically, Glenn et al. (2017) [18] reported that a moderate dose of CM (8 g) enhanced total repetitions completed on leg press and flat barbell bench press at 80% 1RM in female recreational athletes. The discrepancies between this study and Glenn et al. (2017) [18] may be due to differences in the exercise protocol and the NO pathway. Glenn et al. (2017) [18] required every set to be performed to volitional exhaustion and included twice as many sets of exercise (six compared to three), and therefore would have caused a greater level of hypoxia and recruitment of type II fibres. As CM ingestion increases NO production, and the latter is associated with greater effects in hypoxic conditions and type II muscle fibres, this could explain the different outcomes in performance for the CM-MOD condition [19,20]. It is plausible to suggest that future research should investigate protocols with greater metabolic stress to see a performance benefit with CM-MOD. However, CM-HIGH could be used for resistance exercise protocols similar to the current study to improve total repetitions completed.

The use of CM ingestion is postulated to improve aerobic energy provision via increased ammonia clearance [21]. Increased ammonia clearance should have lowered blood lactate in the current study [4]; however, no effects on lactate were observed with either dose in the current study and therefore this acting mechanism cannot be confirmed. Equally, ingestion of CM has been suggested to increase oxaloacetate, as it is dehydrogenated into this compound, and improve the fluxes in and out of the TCA cycle [3]. The current study, however, reported no significant differences between PLA and either CM dose for blood lactate concentrations. Reasons for this are likely due to the intensity of the exercise protocol in the current study, in that it may have been too anaerobic in nature to observe aerobic benefits, as post-exercise lactate was generally high (~10 mmol·L^−1^) [4]. This might also explain why ergogenic benefits were observed with CM in the longer-duration and lower-intensity protocol used by Wax et al. (2015) [6]. These findings, combined with the findings of Glenn et al. (2017) [18], may suggest that the intensity threshold determines the performance outcome following CM ingestion.

The minor increase in total repetitions performed with CM-HIGH may have implications for training by increasing the mechanical tension and metabolic stress. Given this was observed for the CM-HIGH dose only, this should be opted for by end users to maximise the changes in observing these effects. Speculatively, the use of CM-HIGH could also lead to increases in angiogenesis as more repetitions are completed in the set. However, a proxy marker for this would be increased post-exercise lactate, which was not observed in the current study. This may have been due to the low number of exercises and total sets in the current study and therefore explain the rather modest effects on total repetitions. Further work should apply CM ingestion to exercise protocols with more sets and exercises, as these effects may become more pronounced under greater fatiguing conditions.

A limitation of this study was that plasma citrulline concentrations were not measured due to financial constraints, which would have provided useful insight into the dose responses. However, based on prior data by Moinard et al. (2018) [13] reporting that all participants had significantly higher citrulline concentrations from smaller doses than used in the current study, we are confident all participants achieved a concentration that would be ergogenic. Admittedly, Moinard and colleagues’ [13] work is based on citrulline and not CM, and we did not assess the effects of CM compared to citrulline alone. We also had a mixed cohort of males and females of different baseline strength levels, which may have impacted the outcome of the study. Females are purported to have a reduced ergogenic effect from nitrate ingestion due to potential differences in muscle fibre type distribution (more type I than type II compared to males) or due to differences in baseline nitrate concentrations (females have typically higher levels than males) [22,23]. Equally, the baseline level of strength was different amongst the groups, and this is something that could be better controlled for in future work. Regrettably, our sample size was not large enough to group results based on sex, and therefore studies with larger sample sizes are required to explore sex differences further. Finally, future research should investigate the effects of CM on training responses to assess the effects on hypertrophic responses.

## 5. Conclusions

This study aimed to investigate the responses of moderate and high CM doses on resistance exercise performance. These findings suggest that 12 g of CM may offer performance benefits to leg press performance. This challenges current dosing protocols that supplement 8 g CM (i.e., CM-MOD) as opposed to 12 g CM (i.e., CM-HIGH). Future work is required to investigate the effects of CM-HIGH on resistance exercise performance with more exercises and sets, as this may increase the ergogenic potential of CM.

## Figures and Tables

**Table 1:** Participant characteristics.

Sex	Age (Years)	Body Mass (kg)	Height (cm)	70% 1RM for Bent over Row (kg)	70% 1RM Leg Press (kg)
Female	23	76	176	35	115
Female	22	67	162	35	115
Female	21	63	158	25	105
Female	24	62	173	25	75
Female	21	67	171	25	75
Female	22	58	167	30	85
Female	21	60	170	25	45
Male	27	91	177	60	175
Male	24	66	172	60	135
Male	23	78	180	60	205
Male	29	86	185	60	175
Male	25	70	168	60	195

1RM = one-repetition maximum; kg = kilograms; cm = centimetres.

## Data Availability

The data presented in this study are available on request from the corresponding author.
